# [Retracted] Honokiol, a phytochemical from *Magnolia spp.*, inhibits breast cancer cell migration by targeting nitric oxide and cyclooxygenase-2

**DOI:** 10.3892/ijo.2026.5904

**Published:** 2026-06-22

**Authors:** Tripti Singh, Santosh K. Katiyar

Int J Oncol 38: 769-776, 2011; DOI: 10.3892/ijo.2011.899

Following the publication of this paper, it was drawn to the Editor's attention by a concerned reader that, for the cell migration assay data shown in Fig. 2A on p. 772, the 'Honokiol Conc. 10 *μ*M' data panels relating to the 4T1 and MCF-7 cell lines contained an overlapping section, such that data which were intended to show the results from experiments performed in different cell lines had been derived from the same original source. In addition, the western blot data featured in Fig. 4A were published at around the same time in a couple of papers in different journals that featured the same authors, wherein the experiments reported were conducted differently compared with the experimental conditions reported in the above paper; both of these papers have subsequently been retracted.

Given the re-use of data that has been identified in the above paper, together with the apparently incorrect assembly of the data in Fig. 2A, the Editor of *International Journal of Oncology* has decided that it should be retracted from the Journal on account of a lack of confidence in the presented data. The authors were asked for an explanation to account for these concerns, but the Editorial Office did not receive a reply. The Editor apologizes to the readership for any inconvenience caused.

